# Recanalization strategies in childhood stroke in Germany

**DOI:** 10.1038/s41598-021-92533-0

**Published:** 2021-06-25

**Authors:** Martin Olivieri, Anna-Lisa Sorg, Raphael Weinberger, Karin Kurnik, Christoph Bidlingmaier, Sabrina Juranek, Florian Hoffmann, Karl Reiter, Michaela Bonfert, Moritz Tacke, Ingo Borggraefe, Florian Heinen, Lucia Gerstl

**Affiliations:** 1 Pediatric Hemostasis and Thrombosis Unit, Department of Pediatrics, Dr Von Hauner Children’s Hospital, University Hospital, LMU Munich, Lindwurmstr. 4, 80337 Munich, Germany; 2 Institute of Social Pediatrics and Adolescent Medicine, LMU Munich, Munich, Germany; 3 Pediatric Intensive Care Unit, Department of Pediatrics, Dr Von Hauner Children’s Hospital, University Hospital, LMU Munich, Munich, Germany; 4 Department of Pediatric Neurology and Developmental Medicine and LMU Center for Development and Children With Medical Complexity, Dr Von Hauner Children’s Hospital, University Hospital, LMU Munich, Munich, Germany

**Keywords:** Neuroscience, Health care, Neurology

## Abstract

Childhood arterial ischemic stroke (CAIS) is a rare event. Diverse etiologies, risk factors, symptoms and stroke mimics hamper obtaining a fast diagnosis and implementing immediate recanalization strategies. Over a period of 3 years (2015–2017), the data of 164 pediatric patients (> 28 days of life-18 years) with a first episode of AIS were submitted to a hospital-based nationwide surveillance system for rare disorders (ESPED). We report a subgroup analysis of patients who have undergone recanalization therapy and compare these data with those of the whole group. Twenty-eight patients (17%) with a median age of 12.2 years (range 3.3–16.9) received recanalization therapy. Hemiparesis, facial weakness and speech disturbance were the main presenting symptoms. The time from onset of symptoms to confirmation of diagnosis was significantly shorter in the intervention group (4.1 h vs. 20.4 h, p ≤ 0.0001). Only in one patient occurred a minor bleed. Cardiac disease as predisposing risk factor was more common in the recanalization group. Recanalization therapies are feasible and increasingly applied in children with AIS. High awareness, timely diagnosis and a large amount of expertise may improve time to treatment and make hyperacute therapy an option for more patients.

## Introduction

Childhood arterial ischemic stroke (CAIS) in children aged older than 28 days of life and up to 18 years is a rare event, with an incidence of approximately 1–3/100,000 children/year^1–3^. Time delay in diagnosing CAIS has been a greatly challenging issue for several years. Increased awareness, implementing pediatric stroke protocols and algorithms has led to faster diagnoses, and therefore, more patients have become eligible for potential recanalization therapies^4–6^. Nevertheless, the diagnosis and treatment of CAIS remains challenging due to the existence of a multitude of stroke mimics and different predisposing risk factors, such as arteriopathy, thrombophilia, cardiac disorders, metabolic disorders or cancer^7,8^. Several case series have demonstrated the feasibility of intravenous thrombolysis, but a prospective randomized trial failed due to recruiting problems (small patient number and presence of contraindications for recanalization therapy (RT))^9^. Moreover, long-term outcome data are lacking^10–12^. In contrast to adult stroke management a different point of view for CAIS is necessary: (a) cerebral arteriopathy is one of the most common causes of CAIS^13^, (b) this arteriopathy may be associated with an increased risk of interventional complication (as dissection, bleeding etc.) (c) distinctive features of developmental hemostasis, such as lower plasminogen (PA) and higher plasminogen activator inhibitor 1 (PAI1) levels in infants, (d) the supposed lower effectiveness of standard recombinant tissue plasminogen activator (rt-PA) doses^10–12,14,15^, and (e) the presumed lower bleeding risk in children than in adults^16^. Since 2015 positive results of several studies for endovascular treatment of adult stroke have raised the question of whether thrombectomy is also feasible in pediatric stroke^17–19^. Additionally, the DAWN^20^ and DEFUSE^21^ trial suggest larger treatment windows in adults and may further increase the number of eligible children with AIS despite longer delay from last time seen well and time of diagnosis.

Although randomized controlled trials addressing the efficacy of such treatment modalities are lacking, several case series have concluded that endovascular procedures are feasible also in children^22–27^.

Based on active surveillance of childhood stroke in Germany published by Gerstl et al.^28^, we aimed to investigate the rate and characteristics of patients treated with recanalization therapies (RTs). In this subgroup analysis, differences in symptoms, time gaps, factors triggering a time gap compatible with a hyperacute RT (within 6 h after the onset of symptoms) and short-term outcome will be outlined.

## Results

Between 01/01/2015 and 12/31/2017, 164 patients with CAIS were reported to ESPED (26). 17.1% (n = 28) of patients with a median age of 12.2 years (range 3.3–16.9) received hyperacute RT with either thrombolysis (n = 10), thrombectomy (n = 10) or both (n = 8). In the recanalization group, more patients were older than five years than in the entire cohort (89% vs. 62%, p = 0.005). Only three patients were younger than five years (3.3, 4.1 and 4.5 years old). Similar to the whole study group, we observed a male preponderance (m:f, 1.8:1) in the subgroup analysis. Twenty-seven patients were Caucasian (one Asian). Table 1 summarizes patient characteristics for the whole group and for the recanalization and non-recanalization groups, and Table 2 shows the detailed overview for each patient in the recanalization group.

**Table 1:** Patient characteristics.

		All patients (N = 164)		Patients who did not undergo recanalization (N = 136)		Patients who underwent recanalization (N = 28)		p-value
		N	n (%)	N	n (%)	N	n (%)	
**General characteristics**								
Sex		164		136		28		
Male			97 (59)		79 (58)		18 (64)	0.54
Female			67 (41)		57 (42)		10 (36)	
Age [in years, at symptom onset]		164	9.1 ± 6.0	136	8.4 ± 6.1	28	12.271 ± 4.2	< 0.01
Caucasian ethnicity		154	142 (92)	127	115 (91)	27	27 (100)	0.12
Stroke, thrombosis or other stroke-related events in family		164	35 (21)	136	29 (21)	28	6 (21)	0.99
**Time**								
Time from symptom onset to confirmation of diagnosis [in h]		82		58		24		
< 4.5 h			37 (45)		16 (28)		21 (88)	< 0.01
4.5 h to < 6 h			2 (2)		2 (4)		0 (0)	
6 h to < 12 h			11 (13)		9 (16)		2 (8)	
≥ 12 h			32 (39)		31 (53)		1 (4)	
**Short-term outcome**								
Death		161	4 (2.4)	133	3 (2.2)	28	1 (3.6)	0.54
At least one neurological impairment		164	86 (52)	136	70 (51)	28	16 (57)	0.58
Symptoms at discharge		164		136		28		
Hemiparesis			69 (42)		55 (40)		14 (50)	0.35
Facial palsy			24 (15)		19 (14)		5 (17)	0.77
Speech disturbance			19 (12)		15 (11)		4 (14)	0.75
Seizure			11 (7)		11 (8)		–	0.21
Cerebellar symptoms			6 (4)		6 (4)		–	0.59
Visual disturbance			4 (2)		4 (3)		–	1.00
**Complications**								
Any complication (e.g. bleeding, cerebral edema, others)		164	3 (1.8)	136	1 (0.7)	28	2 (7.1)	0.07

Patient characteristics for the whole group (n = 164) and the subgroups of patients that did not (n = 136) and did undergo recanalization therapy (n = 28).

Quantitative variables are expressed as mean ± standard deviation. Categorical variables are expressed as n (%). Test for differences between patients with recanalization vs. patients without recanalization: p-values are obtained from chi-square or fisher exact tests for categorical data and from Wilcoxon rank sum test for continuous variables.

**Table 2:** Individual patient characteristics.

Nr	sex	Age [in years]	Onset of symptoms^+^	State of consciousness at admission	Onset of symptoms to confirmation of diagnosis [in hours]	Symptoms	Risk factor categroies	RT*	Affected vessels	Complications	Short-term outcome^x^
1	Female	15.8	A		4	Hemiparesis, headache, facial weakness, speech disturbance	others	L + T	Middle cerebral artery		H
2	Male	8.6	U	Coma	3	Hemiparesis, headache, decreased consciousness level	Cardiac Arteriopathy	T			H
3	Male	3.3	AB		1.2	Hemiparesis, facial weakness	Cardiac	L + T	Middle cerebral artery		H, F
4	Female	14.1	A	Stuporous No orientation	3.3	Hemiparesis, headache, facial weakness, decreased consciousness level, speech disturbance	Prothrombotic state Migraine	L + T	Middle cerebral artery Posterior cerebral artery		H, F
5	Male	15.4	A	Dizzy No orientation	2.8	Hemiparesis, facial weakness, decreased consciousness level, speech disturbance, vertigo	Cardiac	L + T			H
6	Female	4.1	A	Stuporous No orientation	9.6	Visual disturbance, headache, seizure (generalized), decreased consciousness level, vomiting/ nausea	Cryptogenic	T			N
7	Male	15.6	AB		3.5	Hemiparesis, facial weakness, speech disturbance	Prothrombotic state	L			N
8	Male	15.9	A		2.6	Headache, ataxia, speech disturbance, dysdiadochokinesis	Cardiac	L	Middle cerebral artery		S
9	Male	9.7	AB		2.2	Hemiparesis, visual disturbance, visual disturbance, headache, facial weakness, speech disturbance	Cardiac	L	Middle cerebral artery		H
10	Female	14.1	A	Dizzy No orientation	2.1	Hemiparesis, decreased consciousness level, speech disturbance	Cardiac	L + T	Middle cerebral artery		NA
11	Female	15	AB	Dizzy	2.8	Hemiparesis, facial weakness, decreased consciousness level, speech disturbance, vertigo	Cryptogenic	L	Middle cerebral artery		S
12	Male	15.1	A			Hemiparesis	Cryptogenic	L			NA
13	Male	14.6	A		2.5	Hemiparesis	Prothrombotic state Arteriopathy	L	Middle cerebral artery		H
14	Male	13	A		1.3	Hemiparesis, headache, facial weakness, speech disturbance	Prothrombotic state	L + T			H
15	Female	5.5	P			Ataxia	Migraine	T			H, F, S
16	Male	16.9	AB	Dizzy	2	Ataxia, decreased consciousness level, vertigo, vomiting/ nausea	Cardiac	L			N
17	Male	10.3	P		28	Paresthesia, numbness feeling	Prothrombotic state Airway infection	T	Posterior cerebral artery		P
18	Female	15.6	A		3.7	Hemiparesis, facial weakness, seizure, speech disturbance	Cardiac Prothrombotic state	T	Middle cerebral artery		N
19	Male	11.4	P	Dizzy No orientation	3	Hemiparesis, facial weakness, speech disturbance, paresthesia	Cardiac	T	Middle cerebral artery		D
20	Male	15.5	AB		1.3	Hemiparesis, facial weakness, speech disturbance	Cardiac	T	Middle cerebral artery		N
21	Male	15.1	AB		1.4	Hemiparesis, facial weakness, speech disturbance	Cardiac	T	Middle cerebral artery		N
22	Female	9.1	AB		3.5	Ataxia, speech disturbance, apraxia	Prothrombotic state	L	Middle cerebral artery Posterior cerebral artery		N
23	Male	13.7	A	Coma No orientation	2.5	Hemiparesis, facial weakness, speech disturbance, splay foot position	Cardiac Prothrombotic state	L	Middle cerebral artery		S
24	Male	6.9	A	Coma	8	Hemiparesis, facial weakness, seizure (focal), decreased consciousness level, speech disturbance	Prothrombotic state Hemato-oncological	T	Middle cerebral artery		H, F, S
25	Male	4.5	AB	Dizzy	1	Hemiparesis, facial weakness, speech disturbance	Cardiac	T	Middle cerebral artery		N
26	Male	16.2	A		2.6	Hemiparesis, facial weakness, speech disturbance	Cryptogenic	L			H
27	Female	15.2	U	Coma		Hemiparesis, headache, neglect	Prothrombotic state	L + T	Middle cerebral artery	Cerebral edema	H
28	Female	10.3	P	Stuporous		Hemiparesis, headache, facial weakness, decreased consciousness level, vomiting/nausea	Cardiac	L + T	Middle cerebral artery	Bleding punction site	H, F

Characteristics of each patient in the recanalisation group (n = 28).

^+^A = acute (within minutes), AB = abrupt (within seconds), P = Progressive (within hours) U = unclear; *RT = recanalisation therapy, L + T = thrombolysis and thrombectomy, L = only thrombolysis, T = only thrombectomy.

^x^H = hemiparesis, F = facial palsy, S = speech disturbance, P = paresthesia, N = none, d = death, NA = not available.

Patient characteristics for the whole group (n = 164) and the subgroups of patients that did not (n = 136) and did undergo recanalization therapy (n = 28).

Similar to the whole study group, hemiparesis (79%), facial weakness (61%) and speech disturbance (64%) were the leading presenting symptoms in the recanalization group. The simultaneous occurrence of all three symptoms was significantly more often present in the RT group than in the non-RT group [15 (62.5%, n = 24) vs 15 (14.4%, n = 104), p = 0.0001]. Additionally, the RT group included facial weakness and speech disturbances as presenting symptoms significantly more often than the non-RT group (p = 0.0003, p = 0.007, respectively) (Table 3). Among patients older than 5 years (n = 107), visual disturbances were more frequent in the recanalization group than in the whole group (p = 0.049).

**Table 3:** Presenting symptoms.

	All patients (n = 164)	Patients who did not undergo recanalization(n = 136)	Patients with recanalization (n = 28)	p-value
**Focal symptoms**	149 (91)	121 (89)	28 (100)	0.11
Hemiparesis	110 (67)	88 (65)	22 (79)	0.16
Facial weakness	52 (32)	35 (26)	17 (61)	**< 0.01**
Speech disturbance	68 (41)	50 (37)	18 (64)	**< 0.01**
Visual disturbance	32 (20)	29 (21)	3 (11)	0.30
Ataxia	24 (15)	20 (15)	4 (14)	1.00
Paresthesia	19 (12)	17 (13)	2 (7)	0.66
Other focal symptoms	29 (18)	25 (18)	4 (14)	0.84
**Nonspecific symptoms**	79 (48)	65 (48)	14 (50)	0.83
Headache	39 (34)	30 (22)	9 (32)	0.25
Decreased consciousness level	34 (21)	25 (18)	9 (32)	0.10
Vomiting/nausea	33 (20)	29 (21)	4 (14)	0.61
Vertigo	21 (13)	18 (13)	3 (11)	1.00
Other nonspecific symptoms	5 (3)	5 (4)	–	0.77
**Seizure**	30 (18)	27 (20)	3 (11)	0.42

Variables are expressed as n (%); p-values from x^2^ test or Fisher’s exact test comparing symptoms of patients with RT and symptoms of patients with non-RT.

Presenting symptoms for the whole group (n = 164) and divided by therapy.

**Figure 1:**
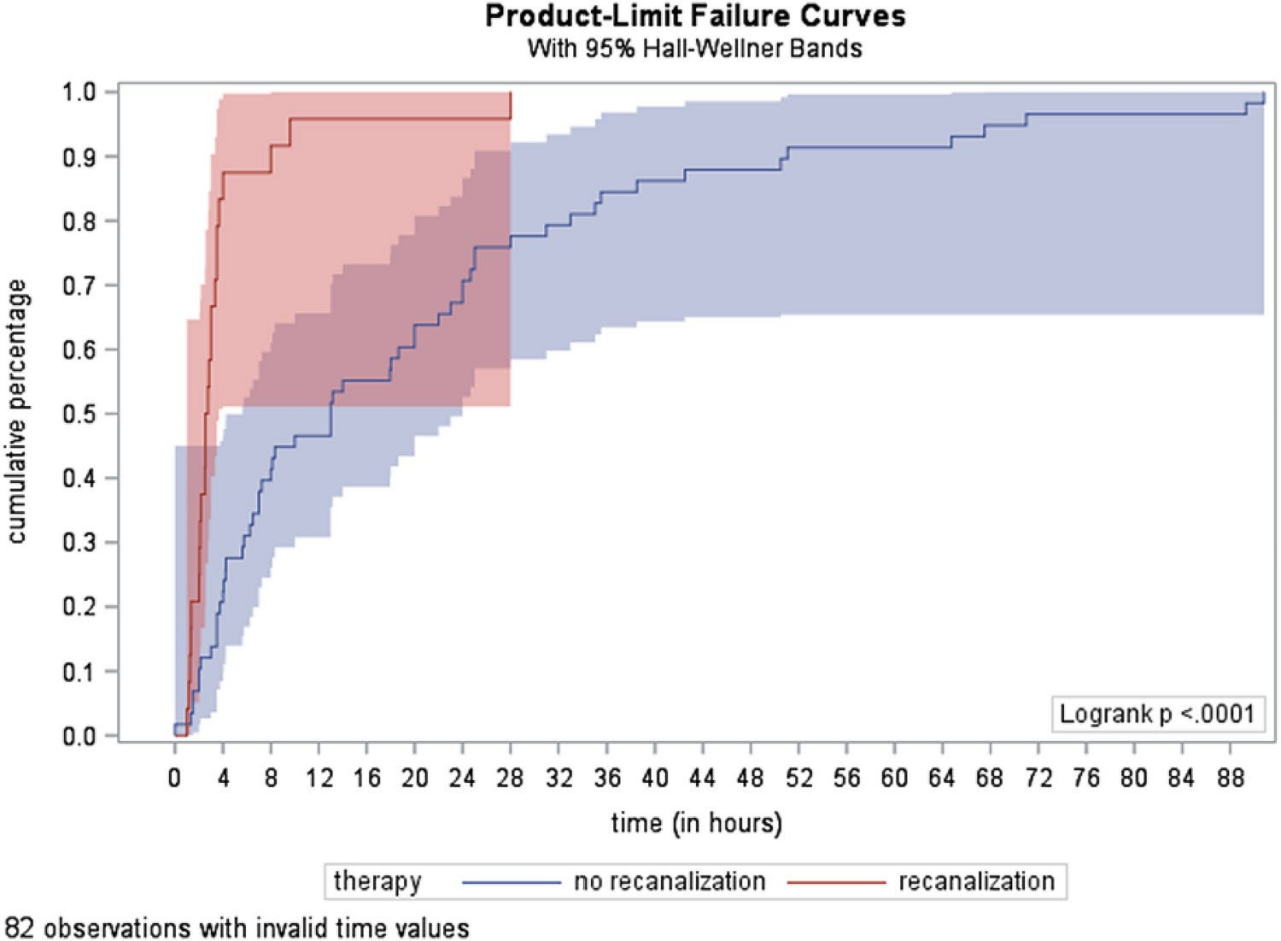
Time from symptom onset to diagnosis. Comparison of the exact time (in h) from the symptom onset to confirmation of diagnosis in the non-RT (n = 28) and RT groups (n = 136).

The time from symptom onset to confirmation of diagnosis was significantly shorter in the RT group than in the group without RT (mean 4.1 h standard deviation (SD) 5.5 h vs. 20.4 h SD 21.8, p ≤ 0.0001) (see Fig. 1; log rank test p < 0.0001). Only in three patients, (age 4.1, 6.9 and 10.3 years) receiving RT diagnosis was confirmed after 6 h (8 h, 9.6 h and 28 h). These patients presented with mainly nonspecific symptoms such as headache, seizure and decreased consciousness. All three patients had a good neurological short-term outcome without any complications.

Cardiac disease as predisposing risk factor was present significantly more often in the recanalization group than in the non-recanalization group (n = 14, 50% vs n = 33, 24%; p = 0.006). The prothrombotic state was equally common in both groups (n = 10, 36% vs n = 46, 34%; p = 0.85), and cerebral arteriopathies were less commonly reported in the RT group (n = 2, 7% vs n = 29, 21%; p = 0.08).

Table 4 lists the affected vessels in both groups, showing that in the RT group, larger vessels, such as the middle cerebral artery (MCA) and internal carotid artery (ICA), were more often affected than in the non-RT group (statistically not significant). In all patients, thrombolysis was performed with recombinant tissue plasminogen activator (rt-PA). Dose information was obtained in 39% (n = 11) of patients. Analogous to adult stroke protocols, the standard dose was 0.9 mg/kg bodyweight. Two patients (both 15 years old) underwent intra-arterial thrombolysis with an absolute rt-PA dose of 20 mg. Only in one patient (11.3-year-old girl) that underwent both thrombolysis and thrombectomy a minor bleed at the puncture site occurred as RT associated complication. No complications occurred in patients treated only with either thrombolysis or thrombectomy.

**Table 4:** Affected vessels.

Affected vessel	All patients (n = 110)	No recanalization (n = 88)	Thrombolysis (n = 7)	Thrombectomy (n = 8)	Lysis and thrombectomy (n = 7)
Middle cerebral artery	75	57	6	6	6
Posterior cerebral artery	20	17	1	1	1
Anterior cerebral artery	15	15	–	-	–
Internal carotid artery	11	9	–	2	–
Cerebellar artery	5	5	–	–	–
Aa. lenticulostriatae	4	3	1	–	–
Basilar artery	2	1	–	–	1
Aa. thalamoperforantes	2	2	–	–	–
A. choroidea ant	2	–	1	–	1
External carotid artery	1	1	–	–	–

Multiple affected vessel per patients (sum larger than total number).

Affected vessels in the RT (n = 22) and non-RT groups (n = 88; data missing from 54 patients).

The short-term outcome defined as residual neurological deficit at discharge was slightly worse in the recanalization group (statistically not significant). At discharge, 57% of the patients in the RT group (vs 51% in the non-RT group) had a least one neurological impairment at discharge. 50% of the patients in the RT group had hemiparesis (vs. 40% in the non-recanalization group), 17% (vs. 14%) had facial palsy and 14% (vs. 11%) had speech disturbance (Table 1). Eight patients in the RT group were discharged without any neurological impairments.

## Discussion

Arterial ischemic stroke in childhood (CAIS) is a rare event but is associated with high clinical, psychological and economic burden due to neurological sequelae^4,29^. Higher awareness among parents, paramedics and health care providers and better diagnostic and treatment modalities seem to have led to an increasing incidence over the last decades. In adults, emerging hyperacute therapies show overwhelming benefits in the treatment and outcome of stroke. Despite faster diagnoses of CAIS, prospective randomized pediatric trials are missing or have had to be stopped due to recruitment problems^9,10^. Nevertheless, a series of observational data confirm the feasibility of thrombolysis and/or thrombectomy in CAIS^9,10,12,23,24,26,30^. The AHA Scientific Statement on the management of stroke in neonates and children as well as the Australian clinical consensus guideline state that the use of hyperacute recanalization treatment remains controversial and should still be limited to some children meeting adult eligibility criteria^6,31^.

Moreover, comparing published pediatric stroke registry data from Amlie-Lefond et al.^12^ (2% recanalization therapy in 4 years, 687 patients) and Bigi et al.^26^ (11% RT in 15 years, 150 patients) with our data^28^ (17% in 3 years, 164 patients), it seems that acute recanalization strategies have been increasingly used over the last years. As demonstrated in our study (88% diagnosed within the supposed critical time window of < 4.5 h), this might be explained by the shorter time intervals from the onset of symptoms to diagnosis and therapy than those in the data published more than ten years ago by Rafay et al. (22.7 h median time interval)^7^. Our data are in accordance with the median time intervals from the onset of symptoms to recanalization (4.0 h) published by Sporns et al.^23^ In the non-recanalization group, more than half of our patients (53%, n = 31) were diagnosed after 12 h. The greater occurrence of symptoms such as speech disturbance, facial weakness and hemiparesis in the RT group might also trigger a faster diagnosis than for nonspecific symptoms^28^. Among patients older than five years, visual disturbances also occurred more often in the recanalization group. This might be associated with problems examining visual disturbances in children younger than five years of age. Three patients (age 4.1, 6.9 and 10.3 years) received recanalization therapy after the recommended time interval with a good short-term outcome. According to adult data from the DAWN and DEFUSE trial a larger therapy window might be supposed also for children and increase the number of patients eligible for RT^20,21^.

More commonly appearing stroke symptoms such as hemiparesis, facial weakness or speech disturbance may explain the significantly higher incidence of thrombectomy in children older than five years. A comparable vessel size of MCA to adults in this patient group facilitates feasibility of RT^32^. Nevertheless, Sun et al. showed that RT might also be feasible in children between nine months and four years of age^33^. Our study adds three more patients below five years of age with successful RT without complications confirming the data from Sun et al. Cardiac risk factors leading to cerebral thromboembolic vessel occlusion were significantly more represented in the recanalization group. Not surprisingly, embolic disease is a mainstay of RT. The incidence of prothrombotic state was similar in both groups, while cerebral arteriopathies were one of the most common risk factors for CAIS associated with a presumed higher interventional risk of bleeding. Relative contraindications such as cerebral arteriopathies, vasospasm or dissection were obviously underrepresented in the RT group.

The observed slightly worse short-term outcome in the RT group might be caused by the small number of patients and a selection bias for individuals being treated by the local treating pediatricians. Additionally, the more severe clinical presentation at start of symptoms, larger affected vessels and/or extension of the infarction area in the RT group could lead to this worse outcome.

### Strength and limitations

The strength of this subgroup analysis is the prospective design of this population-based study^28^. This real-world experience highlights relevant data on the current treatment practice and unmet needs of pediatric stroke care in Germany. The lack of a sufficient number of patients for performing meaningful statistics is a limitation of this study. As mentioned, underreporting to the ESPED may also be a significant limitation. As found by Gerstl et al.^28^ the reported lower incidence of CAIS (0.41/100,000 in the study vs 1–13/100,000 in published data^28^) is due to adult stroke units not reporting adolescents with AIS to the ESPED and in nearly 35% of children generally not treated in children’s hospital^34^. Reports from centers with higher pediatric stroke expertise might explain the low complication rates in this study group. Given the similarity to previously published data, it is unlikely that this underreporting might have influenced the results^12,23,26^. The study design (ESPED survey) without specifying standardized diagnostic and therapy protocols cannot provide further data on long-term outcomes.

## Conclusion

The use of recanalization treatments in CAIS increased over recent years, which might indicate that early recognition of stroke and greater awareness of different treatment options enables the use of RT also in specific patients. Low complication rates in different studies showed their feasibility and safety despite the restrictive recommendations of international pediatric stroke guidelines. The presence of predisposing risk factors such as arteriopathies and low pediatric expertise make it difficult to extrapolate adult data. Pediatric stroke protocols and interdisciplinary treatments in pediatric stroke centers are necessary to identify eligible patients early as possible. Prospective studies on long-term outcomes are needed.

## Methods

The `Erhebungseinheit für seltene Pädiatrische Erkrankungen’ (ESPED) is an established surveillance system for rare disorders in pediatrics. It requires anonymous reporting of rare disorders in childhood on a monthly basis from all German children´s hospitals^35^. The case definition includes any patients with a first onset of CAIS between 28 days of life and 18 years, excluding (presumed) perinatal/neonatal stroke, hemorrhagic stroke and cerebral venous sinus thrombosis. The local treating physicians provided diagnostic modalities, diagnosis and therapy. A pseudonymized, standardized questionnaire was sent to the reporting clinician. Age, onset of symptoms, time span until diagnostic imaging and final diagnosis, imaging technique leading to diagnosis, risk factors and therapeutic strategies were queried^28^. All answer sheets were verified by a pediatric neurologist (LG) and pediatric hemostasis specialist (MO). Data were entered into a web-based database. This subgroup analysis investigates patients treated with intravenous/intraarterial thrombolysis and/or thrombectomy for hyperacute recanalization of affected vessels.

A description of patient’s characteristics was obtained for the entire patient group and by type of treatment irrespective of missing values. Gerstl et al. showed that Pediatric NIH Stroke Scale (PedNIHSS) and the Pediatric Stroke Outcome Measure (PSOM) were barely used in Germany^28^. For this reason, in our subgroup analysis short-term outcome was defined as residual neurological symptoms at discharge. Statistical comparisons of patients with recanalization and patients without recanalization were performed using chi-squared or Fisher exact tests as appropriate. Investigations of the differences in time from the onset of symptoms until a confirmation of diagnosis was based on survival analysis using the product limit estimator. Statistical differences in the time to diagnosis were analyzed with the log-rank test. We used a significance level of 5% for all analyses without adjustment for multiple testing. All statistics were calculated using SAS, version 9.4 (SAS Institute Inc., Cary, NC, USA). All analyzed data involving human participants were in accordance with the ethical standards and with the 1964 Helsinki declaration and its later amendments or comparable ethical standards. The ethics committee waived for informed consent from participants and/or their legal guardians to use patient data to guarantee anonymous incidence reporting of all German cases in accordance with the inclusion criteria and reporting of retrospective pseudonomized data to an independent ESPED data trustee. The data protection office and the ethics committee of the medical faculty of Ludwig-Maximilians University, Munich, approved the study (Nr 42-15; 05-04-2016).

### Statistical analysis

Conducted by Anna-Lisa Sorg and Raphael Weinberger, Institute of Social Pediatrics and Adolescent Medicine, LMU Munich, Germany.

## Data Availability

Study protocol, statistical analyzes and anonymized data will be shared by request from any qualified investigator for the sole purpose of replicating procedures and results presented in the article and as long as data transfer is in agreement with adherence to the legal requirements of Germany and the European Union legislation on the general data protection regulation.
